# Gaps Between Aims and Achievements in Therapeutic Modification of Neuronal Damage (“Neuroprotection”)

**DOI:** 10.1007/s13311-015-0348-8

**Published:** 2015-03-14

**Authors:** Heinz Wiendl, Christian Elger, Hans Förstl, Hans-Peter Hartung, Wolfgang Oertel, Heinz Reichmann, Stefan Schwab

**Affiliations:** 1 Department of Neurology, Albert-Schweitzer-Campus 1, Gebäude A 1, 48149 Münster, Germany; 2 Department of Epileptology, University of Bonn Medical Centre, Bonn, Germany; 3 Klinik für Psychiatrie und Psychotherapie TU München, München, Germany; 4 Department of Neurology, Heinrich Heine University Düsseldorf, Düsseldorf, Germany; 5 Klinik für Neurologie, Philipps-Universität Marburg, Marburg, Germany; 6 Klinik und Poliklinik für Neurologie, Universitätsklinikum Carl Gustav Carus Dresden, Technische Universität Dresden, Dresden, Germany; 7 Neurologische Klinik, Universitätsklinikum Erlangen, Erlangen, Germany

**Keywords:** Neuroprotection, neurodegeneration, Alzheimer’s disease, Parkinson’s disease, multiple sclerosis, stroke

## Abstract

The term “neuroprotection” is often misused, overused, or misunderstood. A reasonable definition of neuroprotection refers to the preservation of “neuronal structure and/or function.” Although our knowledge about the cellular and molecular mechanisms of neurodegeneration has expanded, experimental systems and animal models that mimic the process or allow translation into clinical success remain limited. This editorial discusses reasons for this gap and strategies to close it. Experimental models can only mirror certain aspects of disease mechanisms in humans. Therefore, findings in these models need to be linked with patient data to improve real-life relevance. Successful neuroprotection depends on finding the right “window of opportunity” which varies from very short (stroke) to very long (Alzheimer’s disease), necessitating the need to focus on strategies for very early disease recognition. This need challenges the strategies to be chosen, trial approaches and methodologies, and the allocation of resources. Additionally, outcome measures are often not well suited to assess neuroprotection. To this end, surrogate measures, including biomarkers, are useful endpoints to demonstrate evidence of target directed therapeutic utility. Finally, studies have shown that neuroprotection is not likely to succeed when targeting only one pathway. These obstacles have reduced the level of enthusiasm for neuroprotection in certain disease areas (e.g., stroke). Academia, industry, regulatory authorities, funding agencies and patient organizations have to cooperate to a greater extent in order to overcome these impediments and to encourage nonclassical concepts. These concepts will be interdisciplinary in order to achieve meaningful disease modification.

**Electronic supplementary material:**

The online version of this article (doi:10.1007/s13311-015-0348-8) contains supplementary material, which is available to authorized users.

Neuroprotection is certainly among the most desirable therapeutic goals in many major neurologic disorders [e.g., multiple sclerosis (MS), stroke, Alzheimer’s disease (AD), Parkinson’s disease (PD), chronic epilepsy). The term “neuroprotection” is commonly understood as a means of preventing neuronal damage over short or longer time periods. Within this, prevention, rescue and repair mechanisms can be distinguished, all having a potential impact on neuronal survival over time. While decades of research efforts have been dedicated to this goal, the overall therapeutic success in various clinical trials across neurologic disease entities is deplorably limited. Even though there are common features among different diseases and pathogenic mechanisms, the definition of clinical “neuroprotection” in each individual condition remains difficult. This editorial considers a number of ideas as to why such a gap exists between the increasing knowledge of molecular and cellular mechanisms of neurodegeneration and neuroprotection in experimental systems and the translation into clinical success. Reasons are manifold and include: 1) major difficulties regarding the standardization of experimental model systems supposed to mimic the pathogenesis of neurologic disorders; 2) missing the window of opportunity for therapeutic intervention; 3) inappropriate or unfocused selection of patient populations (often combining rather heterogeneous groups mostly based on mere clinical but not biological criteria); 4) inappropriate choice of outcome measures (Appendix Table); 5) traditional clinical trial designs and non-adaptive methodologies; and 6) lack of sufficient funding as neuroprotective trials, in general, last years longer than the standard period of a pivotal registration trial for symptomatic therapies in the abovementioned disorders.

While major progress in the definition of molecular or cellular markers has been made, finding the appropriate window of opportunity for clinical trials has turned out to be a formidable task. This notion has long been obvious in diseases with underlying short-acting, hyperacute, or acute neuronal damage as neurotrauma. Closure of a therapeutic window may also be a critical determinant in chronic neurodegenerative disorders such as AD and PD. Converging evidence suggests an onset of pathological processes years or decades before the manifestation of the first subtle clinical signs and symptoms emerge. In this circumstance, attempts at therapeutic neuroprotection are then initiated too late for an effective disease modification.

In MS, immune-mediated inflammation and neurodegeneration are key pathologic features. Their contribution to disease pathology and outcome differs between stages and phases of the disease. Early (and earliest) interventions with several immune-therapeutic agents in relapsing forms of MS have shown significant benefit with regard to disease modification by reducing inflammatory activity (relapse rates, evidence of inflammatory lesion activity on imaging studies) and in parallel aspects of neurodegeneration (e.g., disability progression, T1 hypointense lesions on magnetic resonance imaging, development of cerebral atrophy) [1, 2]. However, trials aimed primarily at modifying disease progression (in primary progressive or secondary progressive MS) have been largely unsuccessful so far. Conceptual “direct” and “indirect” neuroprotective approaches need to be distinguished, the latter representing the dominating feature of current immune therapies.

Taking the various aspects and experiences into consideration, perspectives and challenges for future trial designs to prove “prevention of neuronal damage” in neurologic disorders appear obvious. Heterogeneity of study populations needs to be better controlled. Integrating paraclinical surrogate measures or biological information (including biomarkers) will help to stratify patients. Additionally, adaptive study designs, less conventional, multiparametric assessments and endpoints that comprehensively and sensitively capture the varied consequences of the neurodegenerative pathobiology on disease activity and progression will have to be considered. Further, multidomain, as well as population-based, approaches with risk-enrichment strategies are required in certain disease areas, leading to rather novel methodological approaches for trial approaches. We recommend substituting the often misconceived term “neuroprotection” with a more pragmatic description such as “prevention of neural damage”.

## How Should we Define “Neuroprotection”?

Very few terms have been more overused, misused, or even misunderstood in clinical neuroscience over the years than “neuroprotection”. Specifically, assumptions as to how this “neuroprotection” could possibly modify the outcome of certain neurologic disorders have been unrealistic. A reasonable definition of neuroprotection refers to the relative preservation of neuronal structure and/or function [3, 4]. More generally, and applied to a variety of neurologic diseases, the aim is to prevent neuronal damage over time (either acute or chronic). The traditional assumption is that the course or outcome of many central nervous system disorders, including disorders associated with acute neuronal damage and chronic neurodegeneration (traumatic brain injury, stroke, MS, AD, PD, etc.), can be modified via exploiting “neuroprotective” mechanisms. Based on current concepts of key effector mechanisms of neuronal damage in the above disorders, experimental treatments often consider targeting oxidative stress, cytotoxicity, or a combination of both, providing direct neuroprotection versus indirect neuroprotection through modification of various collateral mechanisms involving inflammation. Finally, the term neuroprotection is, unfortunately, often misused by industry marketing strategies for advertising agents, with rather questionable potential. From the pragmatic point of view of patients and physicians, “neuroprotection” means keeping neuronal and glial damage under the threshold of symptom manifestation.

## How do we Standardize “Neuroprotective” Approaches and Outcomes of Experimental Models Mimicking Aspects of Neurological Diseases?

Experimental models of neurodegenerative disorders (models of stroke, MS, experimental autoimmune encephalomyelitis, AD, PD, epilepsy etc.) always reflect only certain aspects of the clinical disease. Scientifically, it is appropriate to make extrapolations and cautiously point out analogies of molecular, subcellular, cellular, or systemic alterations in experimental models to human disorders. However, comparability and standardization of experimental model systems across laboratories is a goal that should be achieved before preclinical data are accepted to be taken into a clinical research program [5]. Even after this problem with experimental systems is resolved with rigorous multicenter standardizations, models can only mirror certain aspects of usually multifaceted disease mechanisms in humans. Several groups are now trying to define criteria for how experimental standards should be adjusted to increase the likelihood of obtaining relevant findings in those models, and to make them more plausible or to translate them into proof-of-concept clinical trials. Obviously, such measures may increase the reliability of experimental models, raising the hope that this may aid in a faithful translation to the clinical realm, but still cannot provide statistical probability of potential outcome success in human diseases. Prudent use of experimental models takes into consideration the notion that these may replicate human disease only partially both in terms of categories of pathobiology and temporal evolution. Therefore, findings in these models need to be linked with patient data to improve real-life relevance. In other words, human data from pathological and nonpathological controls need to be integrated in order to evaluate and potentially validate findings in animal models.

## Are we Approaching the Right “Window of Opportunity” in the Target Diseases?

Epidemiologic, pathophysiologic, and clinical trial data have clearly demonstrated that there are limited windows of opportunity or time lines for the therapeutic modification of neuronal damage, which are specific for every disease. The best-known scenarios for acute diseases with a very small window of opportunity are stroke, and traumatic brain and spinal cord injury. Here, myriads of unsuccessful trials of applying neuroprotection have emphasized the existence of and the need for defining a “window of opportunity” (e.g., thrombolysis). Agents presumed to interfere with such acute/hyperacute mechanisms need to be administered as early as possible to attain efficacy. It still remains doubtful whether approaches targeting single pathways or molecular structures are at all capable of influencing such a dramatic cascade of multiple events leading to acute cell death due to the hypoxic/excitotoxic nature of the acute event. Here, neural repair and strategies to promote neuronal plasticity after acute damage are certainly complementary to the primary aim of preventing neuronal damage. Naturally, disorders such as AD and PD need to be considered on the other, chronic, end of the spectrum. Prodromal phenotypes—such as rapid eye movement sleep behavior disorder in the case of PD or social withdrawal in the case of AD—can be observed before the threshold for the classical clinical syndrome is reached [6–8]. Therefore, we need to think more in terms of very early recognition, intervention, or even prevention strategies. These would impose enormous challenges on strategies to be chosen, trial approaches, and methodologies to be implemented and, therefore, risk taking on the allocation of available resources of scientific personnel and funding. In MS, several examples have now clearly demonstrated that highly active, anti-inflammatory agents (e.g., the monoclonal antibodies natalizumab and alemtuzumab, or new immune therapeutic small molecules such as the first-in-class sphingosin 1 phosphate receptor modulator fingolimod) are quite effective in influencing disease activity, both in terms of relapse frequencies and disability progression. The therapeutic efficacy has even prompted definition of new outcome measures such as “no evidence of disease activity”, “no evidence of disease activity including ongoing atrophy”, or “freedom of disease” [9]. However, in trials of primary and secondary progressive MS, strategies arguably work in relapsing forms of disease show—to a certain extent—reduction of inflammatory aspects of the disease but no clinically significant improvements on disability progression as a measure of ongoing and cumulative neurodegeneration [10, 11]. This clearly advocates the concept of a “window of opportunity” to prospectively prevent progression in MS. It does not resolve the problems of individuals who have started as primary progressive or who are entering the phase of secondary progression, despite therapeutic interventions with drugs for the relapsing remitting phase of the disease [12].

## Are we Using Appropriate Outcome Measures?

Recent years have shown that the choice of primary and secondary end points for several diseases in respective clinical trials is challenging [13]. Regulatory authorities are often rigid in their views regarding which outcome measures have to be used and how. A standard requirement of importance relates to sensitivity to capture change and whether this is clinically meaningful. It is true that some of these outcome measures are hard to standardize, quite reductionist, sometimes almost 1-dimensional. Efforts to establish accepted alternative outcome measures and demonstrate their increasing utility in the absence of comparative studies have followed a rocky path. An example in place is the expanded disability status scale (EDSS) in MS, the most commonly used score to assess disability and its progression. Despite the obvious and multifold limitations (including high inter-rater variability, ordinal nature, and the limited focus on walking capability above values of 4.0), none of the alternative measures has reached a reasonable level of acceptance and broad usage in the MS trial community. This leads to continued use of and reliance on EDSS as the preferred outcome measure to assess disability in MS. MS has seen undisputed success stories over the last 2 decades, but also numerous failed trials of agents aiming at targeting neurodegeneration [1, 14, 15]. It should be noted that clinical trials have strict diagnostic criteria that are often dependent on elements characteristic of late disease, a clear impediment to studying neuroprotection. New vistas and ways to measure neuroprotection are necessary. Surrogate measures, including imaging markers (magnetic resonance imaging, positron emission tomography, single-photon emission computed tomography), biomarkers from different biocompartments, or different stratification approaches (biological, genetic, clinical, etc.), are often not integrated or considered. Specifically, with regard to proof-of-concept assessments, more domains and adaptive, flexible trial designs are necessary for better stratified approaches. Some of the more modern phase II trial designs have been or are developed in this direction [16, 17]. It should be acknowledged that confounding environmental and genetic factors have a high impact on neuroprotection but are very hard to define and/or to control. The growing field of different “omics” and system biology approaches will provide possible algorithms to integrate this knowledge into therapeutic approaches. One key issue will be to integrate regulatory authorities closely in the process of developing new end points for “neuroprotection trials”.

Taking MS as a paradigm, lessons for the future also concern the limitations of included study participants: many studies simply suffer from an enormous heterogeneity of the patient population enrolled, mostly based on rather superficial clinical criteria, while neglecting additional underlying biological surrogate information. To achieve more homogenous cohorts, inclusion information should be broader and supplemented by surrogate and/or biomarkers (e.g., cerebrospinal fluid, peripheral blood, optical coherence tomography, etc.). Also, with regard to trial design, realistic assumptions have to be made in terms of power calculations, trial durations (at least 36 months in MS for meaningful phase III trials to assess influence on neurodegeneration). For phase I and phase II trials, surrogate measures should certainly be allowed as helpful end points to demonstrate evidence of target directed therapeutic utility.

## Challenges, Approaches and Perspectives for Future Trial Designs to Measure Brain Protection or Prevention of Brain Damage

As alluded to above, multiple clinical trials aimed at protecting neurons and the neural parenchyma in neurologic disorders have more often failed than provided evidence for therapeutic efficacy. The reasons for this are numerous, but especially regarding trial design and choice of outcome measures, a number of lessons have been learned and should guide future drug developments [13]. While nobody will disagree that clinical outcomes with patient-relevant measures are certainly most achievable, a number of these outcome measures lack both sensitivity and standardization. Integration of clinical and paraclinical measures might be one way to overcome these limitations. At least objective surrogates may help to better understand the influence of therapeutic interventions on assumed pathogenic mechanisms and measurable outcomes. Although there are certainly commonalities among different diseases with respect to the difficult aim of assessing brain protection and preventing brain damage, major differences exists in their underlying pathogenic mechanisms, disease courses, and individual patient factors. Therefore, only certain aspects within a harmonized approach to achieve brain protection in several neurologic disorders can be generalized, such as the call for standardization and better evaluation of experimental models mimicking aspects of the disease. In terms of clinical trials, it is required to define both the standards of outcome measures, the integration of modern and traditional surrogate measures, and the sequence of phase I to phase III (with partly adaptive) trial designs. Very chronic neurodegenerative disorders, especially AD and PD, probably have a higher likelihood for success with extremely early, biomarker-based, or even preventive, strategies using risk-enrichment selection criteria (e.g., family, genetics, biomarkers, prodromal symptoms). For MS, a field that has highly benefitted from recent repeat successes of therapies targeting inflammation, the unmet need to affect disease progression, as the most likely consequence of continuing neural damage, remains unresolved. Recently, a large trial in primary progressive MS using fingolimod, a substance working both in the peripheral immune system and in the central nervous system [18, 19], has shown no clinical benefit in a modern, multiparametric, multidomain outcome measures trial over 3 years [20]. The reasons for this failure remain to be determined. One general conclusion would be that there is a low likelihood of success targeting one pathway or one selective mechanism within a rather heterogeneous, nongenetically determined condition operative over many years. One example for such a “failed” strategy is the definition of secondary progression as a homogenous disease stage of MS. Secondary progression may have various reasons: it may result from multiple preceding, interlinked, or noninterlinked, dependent or independent pathogenic mechanisms. The term eventually leads into a “melting pot” of progressive disease forms with different “flavors” [21]. This would raise the possibility that in progressive forms of MS combination treatment approaches could attain a more prominent role, addressing and tackling several bona fide candidate pathways at the same time.

In summary, prevention of neuronal damage (“neuroprotection”) remains the holy grail of disease modification in several major neurologic disorders. Recent years have clearly diminished the level of enthusiasm for direct neuroprotection in certain disease areas (e.g., stroke) but have refined the definition of treatment goals, which prevent neuronal damage, either directly or indirectly. The emerging field of elucidating and better defining disease heterogeneity with the integration of surrogate markers, biomarkers, genetics, epigenetics, and system biology approaches in conjunction with novel clinical trial designs and methodologies currently open up new avenues. Within this “prevention strategies”, risk enrichment, stratification, and selection strategies also apply. Academia, industry, regulatory authorities, funding agencies, and patient organizations have to join forces in order to encourage novel, nonclassical, and nonconventional concepts. The spectrum expands from nearly population-based approaches for gathering cohorts, and possibly integrating multidimensional clinical, genetic, environmental (including nutritional) information (at the expense of controlled information at all levels) to highly selected and stratified smaller patient groups with robust, high-resolution parallel biological information. Those novel approaches can help to initiate a new era of clinical studies, different from prototypic double-blind, placebo-controlled trials. A recent encouraging example is represented in the field of AD: individuals without any disease symptoms but genetically predisposed (two copies of the APoE4 gene) will test 2 drugs targeting the amyloid protein [22].

Finally, it is instrumental and most helpful, if not essential, to integrate the expertise and experiences from different fields of clinical neuroscience within interdisciplinary panels. This clearly helps to avoid “blind sides” and thus overlooking lessons that others already learned from failed or successful approaches.

### Electronic supplementary material

Below is the link to the electronic supplementary material.

ESM 1 (PDF 1225 kb)

## Appendix

**Table 1:** Established and promising end points for measuring neuroprotection in multiple sclerosis trials

Promising and potential end points	
Clinical end points	
	Disability progression on Expanded Disability Status Scale (EDSS)
	Multiple Sclerosis Functional Composite (MFSC)
	Paced Auditory Serial Addition Test (PASAT) component of MSFC
	Symbol digit Modalities Test (SDMT)
	Quality of life 36-item Short Form (SF36) Healthy Survey, Multiple Sclerosis Quality of Life Inventory (MSQLI) and NeuroQual
	**Brain and spinal cord imaging parameters**
	T1-hypointense lesion volume
	Time to evolution of T1 gadolinium-enhancing lesions + lesions to T1-hypointense lesions
	% Brain volume change (PBVC)
	% Change in brain parenchymal fraction (BPF)
	% Change in normalized cortical volume (NCVCH)
	Change in gray matter volume
	Change in magnetization transfer ratios in normal-appearing white (NAWM) and gray matter (NAGM)
	Spinal cord atrophy
	Change in brain spectroscopy [*N*-acetyl-aspartate (NAA) and glutamate NAWM and chronic lesions)]
	Nerve fiber changes on diffusion tensor imaging (DfMRI) and diffusion functional magnetic resonance imaging
	Cortical lesions, double inversion recovery (DIR) and phase-sensitive inversion recovery (PSIR)
Ocular end points	
	Peripapillary retinal nerve fiber layer (RNFL) thickness on optical coherence tomography
	Total macular volume (TMV)
	Ganglion cell-inner plexiform layer (GCIPL) thickness on OCT
	Multifocal visual evoked potentials (mfVEP)
Additional biomarker assessments	
	Neurofilament biomarker in cerebrospinal fluid (CSF)

Adapted from [1]
